# Metabolic signature of breast cancer cell line MCF-7: profiling of modified nucleosides via LC-IT MS coupling

**DOI:** 10.1186/1471-2091-8-25

**Published:** 2007-11-29

**Authors:** Dino Bullinger, Hans Neubauer, Tanja Fehm, Stefan Laufer, Christoph H Gleiter, Bernd Kammerer

**Affiliations:** 1 Department of Pharmacology and Toxicology, Division of Clinical Pharmacology, University Hospital Tübingen, Otfried-Müller-Str. 45, 72076 Tübingen, Germany; 2 Department of Obstetrics and Gynecology, University Hospital Tübingen, Calwerstr. 7, 72076 Tübingen, Germany; 3 Institute of Pharmacy, University of Tübingen, Auf der Morgenstelle 8, 72076 Tübingen, Germany

## Abstract

**Background:**

Cancer, like other diseases accompanied by strong metabolic disorders, shows characteristic effects on cell turnover rate, activity of modifying enzymes and DNA/RNA modifications, resulting also in elevated amounts of excreted modified nucleosides. For a better understanding of the impaired RNA metabolism in breast cancer cells, we screened these metabolites in the cell culture supernatants of the breast cancer cell line MCF-7 and compared it to the human mammary epithelial cells MCF-10A. The nucleosides were isolated and analyzed via 2D-chromatographic techniques: In the first dimension by cis-diol specific boronate affinity extraction and subsequently by reversed phase chromatography coupled to an ion trap mass spectrometer.

**Results:**

Besides the determination of ribonucleosides, additional compounds with cis-diol structure, deriving from cross-linked biochemical pathways, like purine-, histidine- and polyamine metabolism were detected. In total, 36 metabolites were identified by comparison of fragmentation patterns and retention time. Relation to the internal standard isoguanosine yielded normalized area ratios for each identified compound and enabled a semi-quantitative metabolic signature of both analyzed cell lines.

13 of the identified 26 modified ribonucleosides were elevated in the cell culture supernatants of MCF-7 cells, with 5-methyluridine, *N*^2^,*N*^2^,7-trimethylguanosine, *N*^6^-methyl-*N*^6^-threonylcarbamoyladenosine and 3-(3-aminocarboxypropyl)-uridine showing the most significant differences. 1-ribosylimidazole-4-acetic acid, a histamine metabolite, was solely found in the supernatants of MCF-10A cells, whereas 1-ribosyl-4-carboxamido-5-aminoimidazole and S-adenosylmethionine occurred only in supernatants of MCF-7 cells.

**Conclusion:**

The obtained results are discussed against the background of pathological changes in cell metabolism, resulting in new perspectives for modified nucleosides and related metabolites as possible biomedical markers for breast carcinoma *in vivo*.

## Background

Since many of the presently applied biomedical markers are not recommended for early diagnosis and therapy surveillance of cancer (e.g. CA-15-3 and CEA in breast cancer) [1], an intensified search for more reliable diagnostic markers is required.

Beside the clinical research for characteristic biomarkers in the genomic profile (genomics) and the expressed protein pattern (proteomics), increasing interest is directed towards the end products of physiological processes represented by different metabolite classes.

The field of metabolomics deals with the fingerprint analysis of biochemical alterations among various metabolic degradation products initiated by diseases, drugs and toxins in a defined biological system [2]. Many of the key metabolic pathways are interconnected and thus perturbations – for instance caused by malignant cancer diseases – in one subset can cause implications on the others. The metabolomic approach in conjunction with its practical appliance for early diagnosis and therapy surveillance in certain diseases is to narrow down the great diversity of possible compounds to those with a huge information content for classification predictions.

Possible targets for a characteristic metabolite fingerprint associated with neoplastic cancer diseases are compounds originating from the cellular RNA metabolism and peripheral biochemical processes.

**Figure 1:**
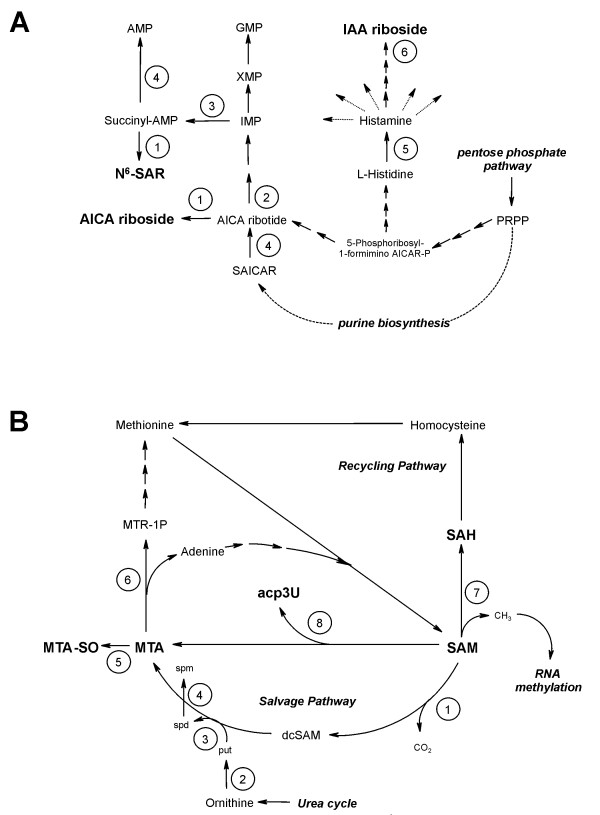
**Cellular pathways interacting with RNA metabolism**. **A: ***purine de novo biosynthesis and histidine metabolism*. Involved key enzymes: 1) 5'-nucleotidase (EC 3.1.3.5), 2) AICAR transformylase (EC 2.1.2.3), 3) adenylsuccinate synthetase (EC 6.3.4.4), 4) adenylsuccinate lyase (EC 4.3.2.2, catalyzes both conversion of succinyl-AMP into AMP and SAICAR (N-succinyl-5-amino-imidazol-4-carboxamid-ribonucleotid into AICA ribotide), 5) histidine decarboxylase (EC 4.1.1.22), 6) phosphatases and 5'-nucleotidase, bold: analyzed metabolites; abbreviations: PRPP (5-phospho-ribosyl-1-pyrophosphate); for all other abbreviations, refer to text. **B: ***polyamine/methionine cycle*. Involved key enzymes: 1) S-adenosylmethionine decarboxylase (EC 4.1.1.50), 2) ornithine decarboxylase (EC 4.1.1.17), 3) propylaminotransferase 1 (EC 2.5.1.16), 4) propylaminotransferase 2 (EC 2.5.1.22), 5) oxygenases, 6) methylthioadenosine phosphorylase (EC 2.4.2.28), 7) DNA-(5-cytosine)-methyltransferase (EC 2.1.1.37), 8) tRNA-uridine aminocarboxypropyltransferase (EC 2.5.1.25),); bold: analyzed metabolites; abbreviations: put (putrescine), spd (spermidine), spm (spermine), dcSAM (decarboxylated SAM), MTR-1P (S-methyl-5'-thio-D-ribose-1-phosphate); for all other abbreviations, refer to text.

The cross-linked pathways of purine de novo biosynthesis and histidine metabolism (Fig. 1A) contribute to the cellular ribonucleotide pool and thus the general build up of RNA molecules. The polyamine/methionine cycle is directly connected to the enzymatic modification of RNA nucleosides via the ubiquitous methyl- and propylaminodonor S-adenosylmethionine (SAM) (Fig. 1B).

RNA contains a series of modified nucleosides in addition to the common ribonucleosides adenosine (A), guanosine (G), uridine (U) and cytidine (C). Modifications like methylation, hydroxylation, reduction, isomerization, sulfur/oxygen substitution and addition of complex side chains are implemented posttranscriptionally within the polynucleotide molecule by various modifying enzyme systems.

The major task of these modifications is thought to be the general improvement of biological activity, integrity and efficiency of RNA in various biochemical processes [3].

At present about 100 modified nucleoside structures are known to occur in different RNA types like transfer RNA (tRNA), messenger RNA (mRNA), ribosomal RNA (rRNA) and small nuclear RNA (snRNA) with tRNA being the most intensively modified (up to 25% in higher eukaryotes) [4].

During posttranslational RNA catabolism, nucleosides are released due to enzymatic hydrolysis of the polynucleotides and subsequent elimination of the phosphate moiety. The common nucleosides C, U, A and G are recycled for intracellular RNA-rebuilding in the so-called salvage pathway by stepwise re-phosphorylation to the corresponding triphosphate nucleotides.

This passage is obstructed for modified nucleosides due to the lack of specific phosphorylases. Thus they are excreted quantitatively out of the cell as metabolic end products via blood stream into the urine [5].

In cancer, RNA metabolism is impaired, which has been demonstrated by altered levels of modified nucleosides. In this context, RNA metabolites have been analyzed in several biological fluids like blood [6] and urine [7] as well as in RNA-hydrolysates [8].

It was shown that the levels of 1-methylinosine (m^1^I) and N^2^,N^2^-dimethylguanosine (m^2^_2_G) are elevated in urine from breast cancer patients [9]. Increased amounts of modified nucleosides were also observed in urine of patients suffering from leukemia [10], gastrointestinal cancer [11] and lung carcinoma [12].

Different methods have been applied for identifying and quantifying nucleosides using high-performance liquid chromatography (HPLC) with UV detection and capillary electrophoresis (CE) [13]. Recently, the coupling of HPLC, gas chromatography or capillary liquid chromatography with mass spectrometric detection via electrospray ionization ion trap mass spectrometry (ESI-IT MS) [14], ESI tandem MS [15] and fast atom bombardment (FAB MS) [16] has been applied. ESI-TOF MS [17] and offline mass spectrometric techniques like MALDI-TOF MS [18] have proven valuable tools for the identification of nucleoside structures.

Bioinformatic methods like artificial neural network (ANN) [19], principal component analysis (PCA) [20], learning vector quantization (LVQ) and support vector machine (SVM) [21] enabled sophisticated nucleoside pattern recognitions, resulting in improved values for specificity/sensitivity and hence a more significant classification in early cancer diagnosis.

Related to metabolic profiling in blood and urine, cell culture supernatants offer the great advantage of the exclusion of subsequent interferences along the excretion pathway, e.g. enzymatic actions in the blood stream, liver and kidney as well as the falsification by bacterial metabolites. Thus, it is possible to generate unaltered metabolite patterns of cellular origin.

**Figure 2:**
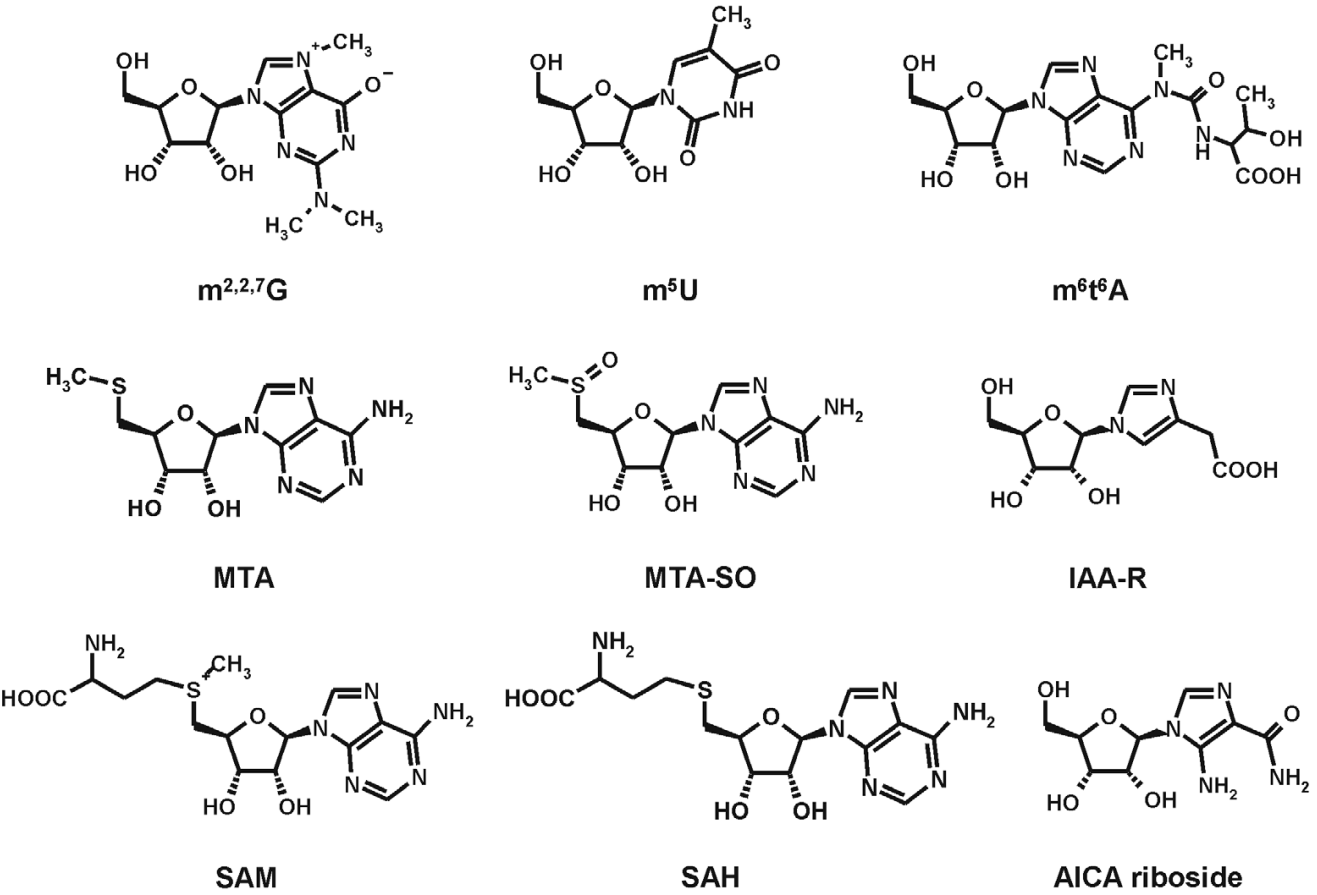
**Structures of some of the identified and analyzed metabolites**. Abbreviations: m^2,2,7^G (N^2^,N^2^,7-trimethylguanosine), m^5^U (5-methyluridine), m^6^t^6^A (*N*^6^-methyl-*N*^6^-threonylcarbamoyladenosine), MTA (5'-deoxy-5'-methylthio-adenosine), MTA-SO (5'-deoxy-5'-methylthioadenosine sulfoxide), IAA-R (1-ribosylimidazole-4-acetic acid), SAM (S-adenosylmethionine), SAH (S-adenosylhomocysteine).

According to the metabolomic approach of contemplating the analyzed biological system as a whole set of more or less interconnected processes, we screened cross-linked metabolic pathways of the cellular RNA metabolism. Therefore we performed a qualitative and semi-quantitative LC-IT MS analysis of nucleosides and related metabolites (Fig. 2) excreted by breast cancer cell cultures to get a deeper insight into properties and variations of cell metabolism at the point of origin.

## Results and Discussion

In the present study, we utilized the potentials of ion trap mass spectrometry to analyze differences in the metabolite excretion patterns of MCF-7 breast cancer cell line and MCF-10A breast epithelial cell line.

**Figure 3:**
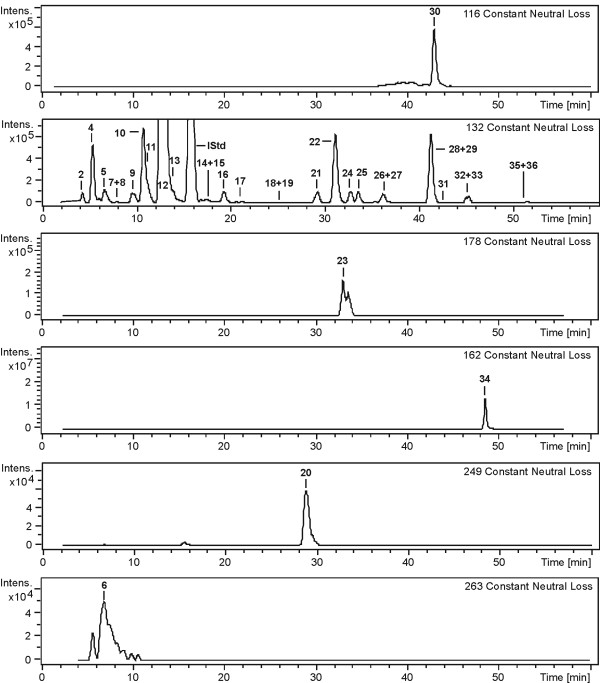
**Constant neutral loss (CNL) chromatograms of a MCF-7 cell culture supernatant sample**. The different CNL amu values are characteristic for the various ribose structures of the analyzed metabolites, lost as a neutral moiety in the MS/MS fragmentation of the labile C-N-glycosidic bond between sugar and base. Numbers refer to metabolites listed in table 1. ψ (no characteristic neutral loss because of stable C-C-glycosidic bond) and IAA-R (not detectable in supernatants of MCF-7) are not shown.

For compound identification we applied auto-LC-MS^3 ^runs [14] of the extracted cell culture supernatant samples. Collision induced dissociation (CID)-experiments enabled the generation of constant neutral loss (CNL) chromatograms, showing only those compounds losing a defined functional group in the first fragmentation step. In nucleoside structures, this step is generally represented by the characteristic decay into the neutral sugar moiety and the corresponding nucleic base fragment at the fragile N-glycosidic bond. Loss of 132 amu is a strong indication for metabolites with an unaltered ribose, 116 amu loss points at a 5'-deoxyribose decay (2'-and 3'-deoxyribose moieties can be excluded due to the requirement of a cis-diol structure for retention on the affinity chromatography column). 5'-deoxy-5'-methylthioadenosine (MTA, CNL: 162 amu), 5'-deoxy-5'-methylthioadenosine sulfoxide (MTA-SO, CNL: 178 amu), S-adenosylhomocysteine (SAH, CNL: 249 amu) and SAM (CNL: 263 amu) eliminate the corresponding thioribose derivatives. (Fig. 3).

Nucleosides with a stable C-glycosidic bond like pseudouridine (Ψ) do not loose the entire sugar moiety in the first fragmentation step but show multistep water eliminations within the ribose structure.

**Figure 4:**
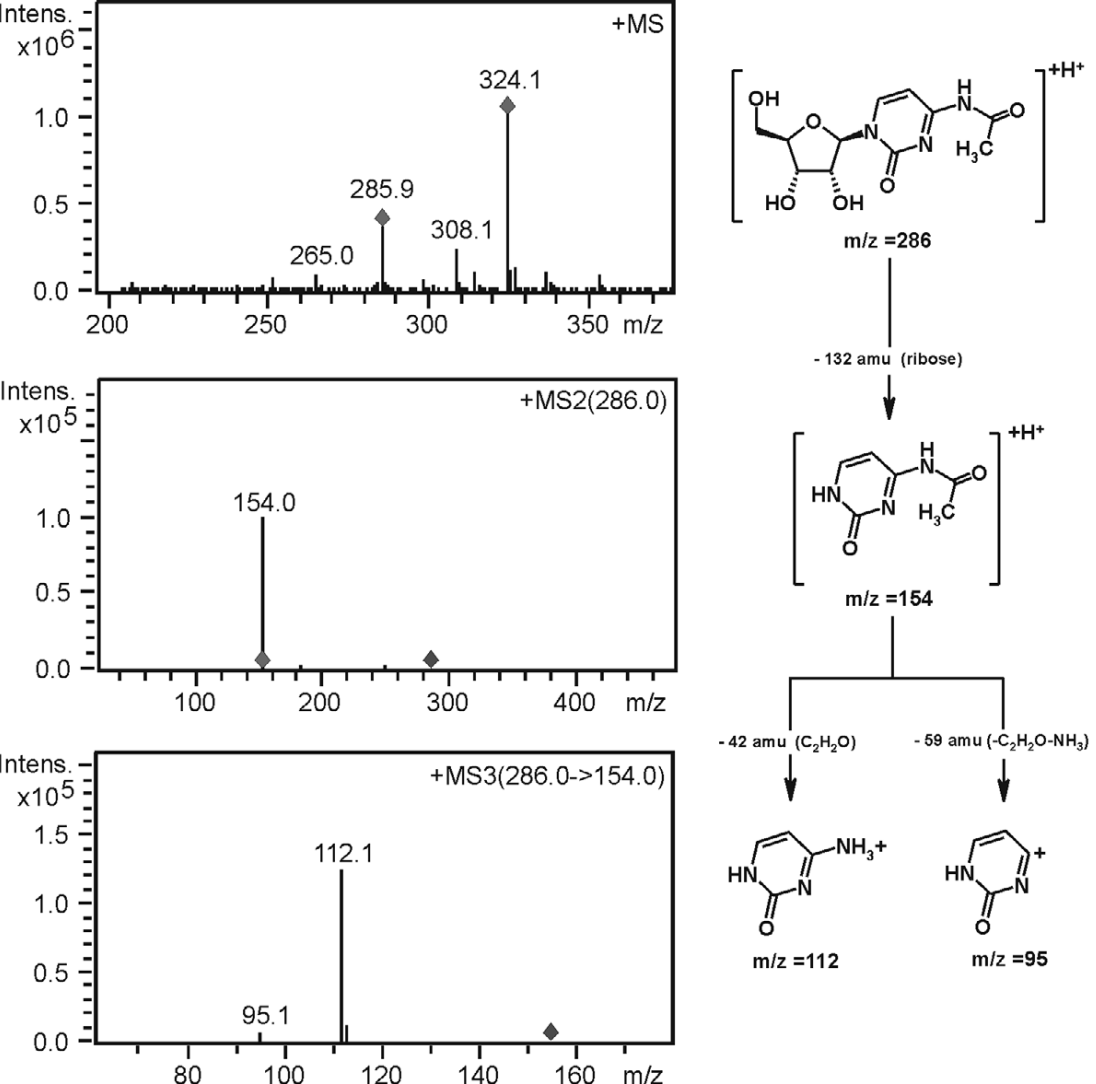
Fragmentation pathway proposal for the modified nucleoside N^4^-acetylcytidine (ac^4^C).

The characteristic decay of the sugar moiety (ribose or modified ribose) observed in the MS/MS step and the subsequent fragmentation of the remaining heterocycle (e.g. the corresponding nucleic base) by MS^3 ^gave precious information about the structural identity of the isolated compounds (Fig. 4).

26 nucleosides and ten additional compounds from different metabolic cell pathways were identified by comparison of fragmentation patterns and retention time to analogous standards.

1-ribosylimidazole-4-acetic acid (IAA-R) and 3-(3-aminocarboxypropyl)-uridine (acp^3^U) were previously identified by our research group in human urine with combined LC-IT MS and ESI-TOF MS measurements [17]. 1-ribosyl-pyridin-2-one-5-carboxamide (2,5-PCNR) was identified by MALDI-TOF MS analysis [18].

1-ribosylnicotinamide (NA-R), 5-carbamoylmethyluridine (ncm^5^U), MTA-SO, 5'-deoxyadenosine (5'-dA), *N*^6^-methyl-*N*^6^-threonylcarbamoyladenosine (m^6^t^6^A), 2-methylthio-*N*^6^-threonyl-carbamoyladenosine (ms^2^t^6^A) and 5-methoxycarbonyl-methyl-2-thiouridine (mcm^5^s^2^U) were identified in a parallel in-depth study of urinary metabolites by accurate mass measurement via FT MS combined with fragmentation pattern analysis by IT MS (data not published yet, paper in preparation). As the obtained fragmentation pathways and retention times of the mentioned compounds are similar in both analyzed biological fluids, they are most probably identical. An assortment of MS^3 ^fragmentation patterns for selected metabolites is shown in Table 1.

**Table 1:** IT MS^3 ^fragmentation patterns of selected metabolites, occurring in the analyzed cell culture supernatants.

**compound name**	**MS**	**MS^2†^**	**MS^3^**
1-ribosylimidazole-4-acetic acid	**259**	**127 **(MH^+^-R)	**81 **(127-HCOOH)
1-ribosylnicotinamide	**255**	**123 **(MH^+^-R)	**106 **(123-NH_3_)
			**96 **(123-HCN)
			**80 **(123-HNCO)
S-adenosylmethionine	**399**	**136 **(MH^+^-mR)	**119 **(136-NH_3_)
			**109 **(136-HCN)
			**94 **(136-NH_2_CN)
5-carbamoylmethyluridine	**302**	**170 **(MH^+^-R)	**153 **(170-NH_3_)
1-ribosyl-4-carboxamido-5-aminoimidazole	**259**	**127 **(MH^+^-R)	**110 **(127-NH_3_)
S-adenosylhomocysteine	**385**	**136 **(MH^+^-mR)	**119**(136-NH_3_)
			**109**(136-HCN)
			**94 **(136-NH_2_CN)
5'-deoxy-5'-methyl-thioadenosine sulfoxide	**314**	**136 **(MH^+^-mR)	**119**(136-NH_3_)
			**109**(136-HCN)
			**94**(136-NH_2_CN)
5'-deoxyadenosine	**252**	**136 **(MH^+^-mR)	**119 **(136-NH_3_)
			**109 **(136-HCN)
			**94 **(136-NH_2_CN)
5-methoxycarbonylmethyl-2-thiouridine	**333**	**201 **(MH^+^-R)	**169 **(201-CH_3_OH)
			**141 **(201-CH_3_OCHO)
N^6^-methyl-N^6^-threonylcarbamoyladenosine	**427**	**295 **(MH^+^-R)	**150 **(295-C_5_H_7_NO_4_)
2-methylthio-N^6^-threonylcarbamoyladenosine	**459**	**327 **(MH^+^-R)	**208 **(327-C_4_H_9_NO_3_)
			**182 **(327-C_5_H_7_NO_4_)

^† ^R: loss of ribose (132 amu), mR: loss of modified ribose

**Figure 5:**
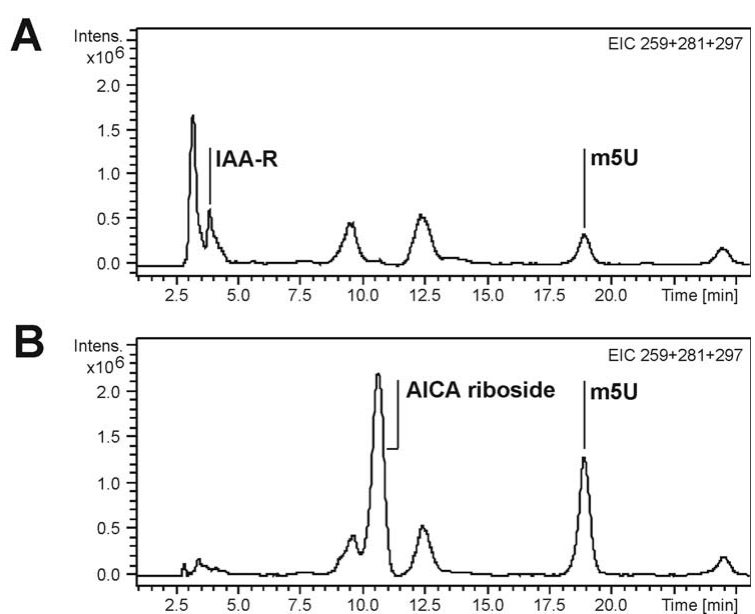
**Characteristic differences in the metabolite excretion of MCF-10A (A) and MCF-7 (B) cell culture supernatants**. Comparison of the Extracted Ion Chromatogram traces (EIC) 259 (M+H+) + 281 (M+Na+) + 297 (M+K+) of the corresponding metabolites IAA-R, AICA riboside and m^5^U from LC-IT MS analysis of cell culture supernatants of mammary epithelial cells MCF-10A and breast cancer cell line MCF-7.

For a comparative, semi-quantitative pattern analysis of the two studied cell lines, the peak areas of the identified metabolites in the processed EICs were related to the peak area of the internal standard isoguanosine (Table 2). Some of the analyzed metabolites show striking varieties between the cancer cell line and the reference cell line, presumably caused by the cellular response to pathophysiological mutations (Fig. 5).

**Table 2:** Metabolite excretion in cell culture supernatants of MCF-10A cells and MCF-7 breast cancer cells.

**No**.	**compound name**	**M+H^+^**	**MCF-10A**	**MCF-7**
			AreaQ *1000^† ^mean (n = 3) ± SD	AreaQ *1000 mean (n = 3) ± SD
1	1-ribosylimidazole-4-acetic acid	259	19.83 ± 1.07	n.d.
2	Dihydrouridine	247	210.30 ± 19.13	261.58 ± 14.58
3	Pseudouridine	245	513.38 ± 26.77	658.02 ± 30.67
4	1-ribosylnicotinamide	255	1985.96 ± 206.67	729.10 ± 43.95
5	Cytidine	244	2044.16 ± 190.68	1066.82 ± 86.71
6	S-adenosylmethionine	399	n.d.	335.55 ± 21.75
7	5-carbamoylmethyluridine	302	32.47 ± 0.73	18.10 ± 0.96
8	3-(3-aminocarboxypropyl)-uridine	346	7.13 ± 0.63	12.89 ± 0.60
9	Uridine	245	1103.56 ± 30.38	504.13 ± 80.28
10	3-methylcytidine	258	382.01 ± 9.65	458.36 ± 16.08
11	1-ribosyl-4-carboxamido-5-aminoimidazole	259	n.d.	251.47 ± 22.35
12	1-methyladenosine	282	2170.75 ± 104.62	2067.45 ± 67.84
13	5-methylcytidine	258	437.16 ± 25.90	446.30 ± 14.00
14	Inosine	269	147.28 ± 9.77	46.89 ± 10.41
15	5-methyluridine	259	35.90 ± 1.48	141.70 ± 4.01
16	Guanosine	284	79.84 ± 8.73	15.31 ± 3.02
17	1-ribosyl-pyridin-2-one-5-carboxamide	271	56.25 ± 27.64	46.70 ± 4.94
18	3-methyluridine	259	20.30 ± 1.25	26.91 ± 0.59
19	Xanthosine	285	13.10 ± 0.27	18.96 ± 1.84
20	S-adenosylhomocysteine	385	7.53 ± 0.43	47.77 ± 0.98
21	1-methylinosine	283	282.12 ± 32.66	269.92 ± 6.25
22	1-methylguanosine	298	648.74 ± 5.15	700.59 ± 10.64
23	5'-deoxy-5'-methyl-thioadenosine sulfoxide	314	23.73 ± 0.95	32.40 ± 6.31
24	N^4^-acetylcytidine	286	296.08 ± 4.84	298.65 ± 6.69
25	N^2^-methylguanosine	298	293.26 ± 14.32	206.55 ± 7.32
26	Adenosine	268	5.97 ± 0.10	32.57 ± 7.16
27	N^6^-succinyloadenosine	384	130.04 ± 2.59	97.78 ± 3.40
28	N^2^,N^2^-dimethylguanosine	312	460.45 ± 19.86	587.62 ± 9.99
29	N^2^,N^2^,7-trimethylguanosine	326	129.55 ± 7.07	263.28 ± 5.54
30	5'-deoxyadenosine	252	101.50 ± 16.49	81.81 ± 2.47
31	5-methoxycarbonylmethyl-2-thiouridine	333	10.55 ± 0.23	12.60 ± 0.34
32	N^6^-methyladenosine	282	22.97 ± 11.04	32.67 ± 12.12
33	N^6^-threonylcarbamoyladenosine	413	42.34 ± 1.83	67.25 ± 0.44
34	5'-deoxy-5'-methyl-thioadenosine	298	3452.12 ± 213.17	5668.00 ± 125.15
35	N^6^-methyl-N^6^-threonylcarbamoyladenosine	427	3.11 ± 0.04	5.96 ± 0.46
36	2-methylthio-N^6^-threonylcarbamoyladenosine	459	3.68 ± 0.15	3.47 ± 0.16

^† ^AreaQ* 1000 = (peak area [M+H^+ ^+ M+Na^+ ^+ M+K^+^] (analyt)/peak area [M+H^+ ^+ M+Na^+ ^+ M+K^+^] (internal standard isoguanosine)) * 1000 (factor), tabulated by increasing retention time, n.d.: not detectable

### Modified nucleosides

We found that the modified nucleosides 5,6-dihydrouridine (DHU), Ψ, acp^3^U, 3-methylcytidine (m^3^C), 5-methyluridine (m^5^U), 3-methyluridine (m^3^U), xanthosine (X), 1-methylguanosine (m^1^G), m^2^_2_G, N^2^,N^2^,7-trimethylguanosine (m^2,2,7^G), mcm^5^s^2^U, N^6^-threonylcarbamoyladenosine (t^6^A) and m^6^t^6^A are elevated in the supernatants of MCF-7 cells compared to those of MCF-10A cells. Therefore, we generally considered a compound level as "elevated" when the area ratio exceeds the mean value of the reference cell line by two standard deviation values (2σ-concept) [5]. The methylated nucleoside N^6^-methyladenosine (m^6^A) is not included in this list because it can be formed through isomerization of 1-methyladenosine (m^1^A) and thus could not be normalized.

Especially the levels of m^5^U with ratios ~4/1 (MCF-7/MCF-10A), m^2,2,7^G (~2:1), m^6^t^6^A (~2:1) and acp^3^U (~2:1) should be pointed out.

m^5^U is present in eukaryotic tRNA and rRNA [22]. Roe and Tsen postulated, that this nucleoside might be involved in the regulation of translational processes in mammalian liver [23]. Its formation in tRNA is associated with the stabilization of the macromolecular structure. A lack of m^5^U, 2'-O-methylguanosine and Ψ at the positions 54, 18 and 55 in tRNAs of *Escherichia coli *induced a reduced growth rate and led to defects in translation processes [24].

m^2,2,7^G is present in eukaryotic mRNA and snRNA [22]. In the latter, the hypermethylated guanosine is part of the m_3_G-cap-structure, a snRNA-specific variant of the regular monomethylguanosine-(m^7^G)-cap at the 5'-termini. Nascent snRNAs (with m^7^G-cap) are transported to the cytoplasm were they assemble with small nuclear ribonucleoprotein particles (snRNPs). Subsequently, the two additional methyl groups are transferred from SAM and the snRNA/protein-complex can enter the nucleus [25].

Small nuclear RNAs belong to a group of non-coding RNAs (ncRNAs). These RNA species have been implicated in diseases including various cancers and neurological diseases [26]. SnRNAs can suppress the expression of genes. They probably bind directly to mRNAs and stop protein production. Additionally snRNAs seem to regulate gene expression epigenetically. The massive amount of ncRNA that is expressed from the genomes of higher organisms suggests that ncRNA may constitute an endogenous control system that regulates the programmed patterns of gene expression [27]. Increased levels of m^2,2,7^G in MCF-7 cells might indicate a "tumorigenic" change of gene expression on the epigenetic level.

To date the occurrence of m^6^t^6^A has been described in tRNA^Thr ^from *E. coli*, wheat embryo tRNA and rat liver tRNA^Ser ^[28]. The methyl group of this nucleoside is supposed to improve the tRNA reading efficiency in *E. coli *[29].

To the authors' best knowledge, this report describes for the first time the identification of this hypermodified nucleoside in human cell lines.

The nucleoside acp^3^U occurs in several mammalian tRNAs [30]. It is posttranscriptionally synthesized via transfer of the aminocarboxypropyl moiety of SAM on certain uridine residues in the RNA macromolecule, yielding MTA as degradation product [31] (Fig. 1B). An elevated production of acp^3^U is thus directly associated with an elevated build up of MTA as observed in MCF-7 cells (see section "MTA and polyamine biosynthesis").

Randerath et al generally proposed that the reason for differences in the base composition of tumor tRNA and thus the excreted modified nucleosides may be changes in the concentrations of specific tRNAs, presence of tRNAs with altered sequences in tumor tissue and aberrant modifications [32].

The methylated nucleosides are synthesized at the posttranscriptional level by transfer of methyl groups from SAM to the nucleobase at certain positions in the RNA molecule by specific enzyme systems. The observed increased excretion of certain methylated nucleosides can be generally traced back to the elevated activity of specific tRNA methylases associated with breast carcinoma [33].

Beside its main task in translational processes, additional functions of tRNA, e.g. the participation as cofactor in enzymatic reactions, have been reported. In this context, tRNA modifications or tRNA modifying enzymes are thought to act as regulatory devices between the translational and other metabolic processes. The degree of modification in the individual tRNA species is regulated differently under the influence of the physiological status of the cell (e.g. the growth rate) [34]. Thus, the observed, elevated excretion of certain modified nucleosides may be attributed to the tumor growth's induced accumulation of specific tRNAs with characteristic nucleoside composition.

The excretion of the common ribonucleosides also shows striking differences between the analyzed cell lines: G, ratio ~1:5 (MCF-7/MCF-10A), U (~1:2), C (~1:2) and A (~5:1). In the latter case, the possible action of adenosine desaminase, converting adenosine to inosine (I) has to be considered (ratio of I: (~1:3)). The phenomenon of a reduced excretion of common nucleosides in MCF-7 might be related to the increased cell turnover and thus RNA metabolism reported in tumor tissue [35] with a higher demand for nucleoside recycling.

### S-adenosylmethionine, S-adenosylhomocysteine

The elevated activity of methylases in malignant cancer diseases has to be estimated in conjunction with the ubiquitous methyldonor SAM and the related metabolic compounds (Fig. 1B).

SAM was solely found in the supernatants of MCF-7 whereas it was not detectable in MCF-10A. Elevated excretion has been observed in cells showing methionine dependent growth [36], like MCF-7.

SAH is the degradation product of SAM when acting as methyldonor in most of the enzymatically induced methyl-transfer reactions, e.g. the methylation of RNA-nucleosides. The inhibitory effect of SAH on methylation reactions is well-documented [37] and explains the elevated level in the cell culture supernatants of MCF-7.

As normally the enzyme systems involved in this pathway are strictly regulated, a severe change in the cell physiology like cancer may lead to an overproduction of SAM for transmethylation and thus to an elevated excretion of SAM and SAH out of the cell.

### MTA and polyamine biosynthesis

MTA and its oxidized metabolite MTA-SO are elevated in the supernatants of MCF-7 cells.

MTA is not a nucleoside derived from RNA but a byproduct in the polyamine biosynthesis. It is generated via transfer of the propylamino moiety from decarboxylated S-adenosylmethionine (dcSAM) on the polyamine-compounds putrescine and spermidine (Fig. 1B). MTA-SO is suggested to arise from MTA by *in vivo *oxidation with peroxides and superoxides or enzymatically by microsomes [38]. Separate studies in our research group confirmed the enzymatically induced oxidation of MTA, yielding MTA-SO by incubation of MTA with rat liver microsomes (data not published). Thus the elevated excretion of MTA-SO in MCF-7 appears to be simply in consequence to the higher MTA level.

Polyamines are regulatory compounds in cell growth processes. Individuals with malignant cancer diseases show elevated amounts of polyamines in physiological fluids [39]. Levels of the enzymes involved in the polyamine biosynthesis (ornithine decarboxylase and S-adenosylmethionine decarboxylase) are higher in tumors than in normal tissue [40]. Thus the polyamine biosynthesis pathway is discussed as useful target for anticancer therapeutics since depletion in polyamine levels has been shown to inhibit cellular growth [41].

The higher excreted level of MTA may be interpreted as a consequence to an elevated polyamine biosynthesis proceeding in rapidly proliferating tumor cells. In human leukemic cells, the relation between the up-regulated synthesis of spermidine and spermine and excretion of MTA has been well-documented [42]. To elucidate this process in MCF-7 cells, the analysis of both intra-and extracellular polyamine levels as well as the determination of the key enzyme activities has to be performed in further experiments.

Another focus is directed towards the metabolic fate of MTA, which is degraded to adenine and S-methyl-5'-thio-D-ribose-1-phosphate (MTR-1P) by the enzyme MTAP (Fig. 1B). In some breast cancer cell lines, including MCF-7, the MTAP gene is inactive [43]. The elevated MTA level in the supernatant of MCF-7 compared to the supernatant of MCF-10A may be explained by this phenomenon, because MTA may not be degraded.

Accumulating MTA is known for its inhibitory effect on polyamine aminopropyltransferase, methyltransferases [44] and cell growth in general [45] – processes important for the growth and development of cancer cells. Thus, the elevated excretion of MTA in MCF-7 may be related to these attributes.

Furthermore, it may be assumed that in case of an inactive MTAP, the methionine synthesis mainly takes place via the recycling pathway. The first step in this cycle is demethylation of SAM to SAH. The elevated levels of both compounds found in the supernatants of MCF-7 support this presumption. The methylation of RNA nucleosides also depends on this cycle, so the increased excretion of methylated nucleosides in the analyzed cell culture supernatants may also be related to the described gene defect.

### Histidine metabolism and purine de novo biosynthesis

A significant difference was observed in the excretion of 1-ribosyl-4-carboxamido-5-aminoimidazole (AICA riboside), the dephosphorylated analog of AICA ribotide (AICAR), which is a crosslinked intermediate in purine *de novo *biosynthesis/histidine metabolism and a precursor of AMP (Fig. 1A).

Whereas AICA riboside was found in the supernatants of MCF-7, it is absent in those of MCF-10A.

A characteristic of tumor cells is the high rate of anabolic processes like lipid-, protein- and DNA biosynthesis. These energy-consuming pathways are regulated by the energy status of the cell, based on the intracellular ATP/AMP ratio. Swinnen et al demonstrated the response of exogenously added AICAR riboside on invasive MDA-MB-231 cells, a well-characterized model of breast cancer. AICA riboside was taken up by the tumor cells and converted to AICAR. This resulted in a distinctive inhibition of tumor-associated anabolic processes, cell proliferation and colony formation as well as an activation of AMP-activated protein kinases (AMPK). At high concentrations applied, apoptosis was induced [46]. AMPKs in term are suggested to be involved in the apoptosis process of cells. An activation as well as a protection against apoptosis has been reported, dependent upon the cell type [47]. It is not known how AMPKs are involved in this process in MCF-7 cells.

However, it may be assumed that tumor cells like MCF-7 excrete endogenous AICAR as its cell permeable analog AICA riboside to avoid the described inhibitory effects on tumor growth and development.

Differences were also observed in the histidine metabolism of the analyzed cell culture supernatants. The histidine metabolite IAA-R is present in MCF-10A supernatants but not detectable in those of MCF-7.

There is evidence suggesting that histamine, the precursor molecule of IAA-R, is of major importance in the development of malignant cancer. An increased enzymatic activity of histidine decarboxylase has been observed in mammary tumor tissue [48]. Functional histamine receptors have been demonstrated in tumor cell lines [49]. Specific inhibitors of the histamine-synthesizing enzyme, histidine decarboxylase, have been shown to inhibit tumor growth in different animal models [50], and *in vitro *[51], suggesting that *de novo *synthesized histamine stimulates tumor growth. An elevated biosynthesis of nascent, tumor-associated histamine is thought to be involved in the maintenance of a maximal growth and proliferation rate by unknown intracellular targets. The mimicking of the polyamine compounds putrescine and spermidine is discussed in this context.

High levels of nascent histamine have also been discussed in relation to a possible stabilization of condensed polynucleotide species, formation of nucleic acid – protein – polyamine complexes, control of aminoacyl tRNA and protein synthesis [52].

Reynolds et al suggested that the high histamine concentration in breast tumors supports the hypothesis that histamine has immunosuppressive effects [53].

The fact that the histamine metabolite IAA-R was solely found in cell culture supernatants of mammary epithelial cells MCF-10A supports the assumption, that the newly synthesized, nascent histamine in MCF-7 is needed for certain processes in the tumor development and thus is not further metabolized in the analyzed tumor cell line.

## Conclusion

In the present study, we applied LC-IT MS analysis as a valid tool for biochemical investigations of altered metabolic pathways in breast cancer cell lines. To the authors' best knowledge, this is the first time that patterns of modified nucleosides and related, ribosyl-conjugated metabolites were analyzed in cell culture supernatants of MCF-7 and MCF-10A cells, both well-defined models in malignant and healthy mammary cells, respectively. We observed remarkable differences in the compared metabolic signatures, concerning modified nucleosides, metabolites deriving from the polyamine/methionine cycle as well as compounds from the purine *de novo *biosynthesis and the histidine metabolism. The discussed metabolic pathways are all involved in the development of malignant cancer diseases.

The obtained data justify a more detailed investigation of metabolic profiles as possible tool to improve prognosis and prediction in diseases like breast cancer. As they are indicators for a changed phenotype in diseased cells, metabolite patterns in body fluids like urine and blood will influence future studies towards a potential non-invasive diagnostic system in early breast cancer.

## Methods

### Cell culture

Breast cancer cell lines were purchased from the American Type Culture Collection (ATCC, Manassas (VA), USA) and cultivation was performed using the recommended media. MCF-7 breast cancer cells (ATCC: HTB-22) were grown in RPMI 1640 basal medium (Biochrome, Berlin, Germany)/10% fetal calf serum (FCS; Biochrome)/1% Penicillin-Streptomycin (Biochrome)/HEPES (Invitrogen, Karlsruhe, Germany). MCF-10A breast epithelial cells (ATCC: CRL-10317) were cultivated in Mammary Epithelial Cell Basal Medium (Promocell, Heidelberg, Germany)/Supplement Pack Mammary Epithelial Cell Growth Medium containing bovine pituitary extract, human epidermal growth factor (EGF; recombinant), bovine insulin, and hydrocortisone (Promocell)/-1% Penicillin-Streptomycin (Biochrome)/Choleratoxin (List Biological Laboratories, Campbell, CA, USA).

In detail, 1 × 10^6 ^cells each were seeded in four plastic flasks and grown in complete medium at 37°C/5% CO_2 _for four days. Then the number of cells was determined by trypsinizing the cells in one flask and counting the viable cells using a counting chamber. In the other three flasks medium was changed to basal medium without additives or fetal calf serum (FCS contains nucleosides itself and would thus lead to false results). After four days of culture the supernatant was collected and cleared from cell debris by applying centrifugal force at 3000 rpm for 10 min. The cleared supernatant was frozen at -20°C until further analysis. The averaged number of cells was equal in both MCF-10A and MCF-7 samples and enabled the direct semi-quantitative comparison of the two analyzed cell lines.

### MCF-7 cell line

The MCF-7 cell line is the most widely used and best characterized of all the human breast cancer cell lines. MCF-7 was isolated from the pleural effusion of a postmenopausal 69 years adult Caucasian woman. The patient had received radiotherapy and endocrine therapy before the appearance of effusion [54]. The estradiol-dependence for growth, antiestrogen sensitivity, and low metastatic potential of MCF-7 cells has led to the hypothesis that they represent an early epithelial adenocarcinoma of the breast [55]. The MCF-7 line retains several characteristics of differentiated mammary epithelium including the ability to process estradiol via cytoplasmic estrogen receptors ER) and the capability of forming domes. Its hormone receptor status is ERα-, ERβ- and progesterone receptor (PR)-positive. MCF-7 cells are an excellent model in which to study the process of malignant progression, because they can be subjected to appropriate endocrinologic and physiologic selective pressures for the derivation of variants with more progressed phenotypes. For example, variants have been selected for antiestrogen resistance. MCF-7 cells lack methylthioadenosine phosphorylase (MTAP) activity, the key enzyme in the methionine salvage pathway [56].

### MCF-10A cell line

Soule et al have described a spontaneously immortalized "normal" human breast epithelial cell line (MCF-10) [57]. These cells were isolated from mastectomy tissue obtained from a premenopausal 36 years old Caucasian patient with fibrocystic disease. After 849 days in culture, a population designated MCF-10A was established. The MCF-10A cells resemble luminal epithelial cells rather than myoepithelial cells, and express antigens for several keratins and epithelial sialomucins [58]. The cells are nontumorigenic in nude mice and do not exhibit anchorage-independent growth. Thus far, the cells have shown no signs of terminal differentiation or senescence. The line is responsive to insulin, glucocorticoids, cholera endotoxin and EGF. Its hormone receptor status is ERα- and PR negative.

### Chemicals

We used methanol LiChroSolv, hypergrade, purchased from Merck/VWR (Darmstadt, Germany) for liquid chromatography. Water was taken from an in-house double distillation system. All other chemicals used were of analytical grade.

The standards used as reference for HPLC separation and compound identification in cell culture supernatants were DHU, Ψ, C, U, A, m^1^A, m^6^A, I, m^1^I, G, m^3^C, 5-methylcytidine (m^5^C), m^1^G, N^2^-methylguanosine (m^2^G), m^2^_2_G, m^3^U, m^5^U, X, N^4^-acetylcytidine (ac^4^C), t^6^A, m^2,2,7^G, MTA, AICA riboside, SAM, SAH, nicotinamide (base standard for MS^3 ^fragmentation comparison with NA-R), imidazole-4-acetic acid (sodium salt, base standard for MS^3 ^fragmentation comparison with IAA-R) and adenylosuccinic acid (sodium salt, base standard for MS^3 ^fragmentation comparison with N^6^-succinyloadenosine (N^6^-SAR).

All standards were from Sigma (Taufkirchen, Germany) except m^2^_2_G, m^2,2,7^G and t^6^A which were obtained from Biolog (Bremen, Germany). The internal standard isoguanosine was kindly donated by Prof. J.H. Kim of Seoul University, South Korea.

Affigel boronate was purchased from Biorad (Richmond, USA).

### Extraction of nucleosides and structurally related compounds from cell culture supernatants

Previous to the HPLC separation, the metabolites were isolated from cell culture supernatants by cis-diol specific affinity chromatography using phenylboronic acid gel. This method was developed by Liebich et al in 1997 [59]. 10 ml of cell culture supernatants were spiked with 100 μl of internal standard solution (0.25 mM isoguanosine), alkanalized to pH 8.8 with 2,5% ammonia solution and then put on the column (500 mg Affigel boronate, column dimensions: 150 × 15 mm). Nucleosides and structurally related compounds are bound reversibly and specifically at the cis-diol group contained in the ribose structure. After washing with 25 mL ammonium acetate solution (0.25 mM, pH 8.8) and 6 ml methanol/water (1:1, v/v), elution was carried out with 50 mL 0.2 M formic acid in methanol/water (1:1, v/v). The solvent was removed using a rotary evaporator and the nucleosides were dissolved again in 0.5 ml ammonium formate solution (5 mM, pH 5). 10 μL of the residues were injected into the HPLC-MS system.

### Instrumentation

The chromatographic separation of the nucleosides was performed on an Agilent 1100 Series HPLC system (Agilent, Waldbronn, Germany) consisting of a Solvent Degasser (G 1379 A), a binary capillary pump (G 1389), an autosampler (G 1313 A), a column oven (G 1316 A) and a DAD (G 1315 B). The chromatographic system consisted of a Merck LiChroCART Superspher 100 RP-18 endcapped column (125 × 2.0 mm i.d., 4 μm (Merck, Darmstadt, Germany)) and a solvent gradient of 5 mM ammonium formate buffer, pH 5.0, and methanol/water (3:2, v/v)+0.1% formic acid. The column was operated at 25°C. The flow rate was set to 125 μl/min using a gradient as described by Kammerer *et al.*[14].

The LC system was coupled to an Ion Trap mass spectrometer. We used a Bruker Esquire HCT-Ion Trap (Bruker Daltonics, Bremen, Germany) equipped with an ESI source in positive ion mode for detection.

The capillary voltage was set to 4 kV, the dry temperature in the electrospray source was 350°C, the nebulizer gas was set to 45 psi and the dry gas to 9.0 l/min. The data were acquired in standard enhanced scan mode (8,100 m/z per second) in a mass range of 200–600 Da.

We performed auto LC-MS^3 ^runs for compound identification by fragmentation pathway and retention time and a LC-MS method without fragmentation processes for semi-quantitative metabolite pattern analysis.

In the latter case, Extracted Ion Chromatograms (EIC) were generated by addition of [M+H^+^], [M+Na^+^] and [M+K^+^] traces to normalize fluctuating alkali-contamination in the samples and the LC-MS system in the course of analysis.

Data were acquired by Bruker EsquireControl version 5.1. For post processing, Bruker DataAnalysis version 3.1 was used.

## Authors' contributions

DB performed the extraction of the cell culture supernatants and the mass spectrometric analysis. HN is responsible for the cell culture work. BK, TF, SL and CG developed concept and design of the study. All authors read and approved the final manuscript.
